# From Symptomatic Treatment to Disease Modification: A Turning Point in Alzheimer’s Disease Management

**DOI:** 10.7759/cureus.47251

**Published:** 2023-10-18

**Authors:** Mahtab Sheikh, Salman J Khan, Haider Ali Tariq Butt, Syed Asjad Tauheed Zaidi, Vineesha NA

**Affiliations:** 1 Internal Medicine, Midland Regional Hospital Portloaise, Portloaise, IRL; 2 Public Health, University of Massachusetts, Amherst, USA; 3 Hematology and Oncology, Mayo Clinic, Jacksonville, USA; 4 Internal Medicine, Midland Regional Hospital Portlaoise, Portloaise, IRL; 5 Medicine, Shalamar Medical and Dental College, Lahore, PAK; 6 Internal Medicine, Liaquat National Hospital and Medical College, Karachi, PAK

**Keywords:** alzheimer's disease, amyloid plaques, lecanemab, novel pharmacotherapy, alzheimer’s dementia

## Abstract

Alzheimer’s disease (AD) is a neurodegenerative disease primarily affecting individuals aged 65 or above. AD leads to progressive cognitive and functional decline, affecting daily life activities. Amyloid plaques are the pathological hallmark of AD, resulting in the loss of neurons and their connections in the brain. For years, patients with AD were treated with pharmacotherapies having only symptomatic effects. Till 2023, no drug was approved for disease-modifying potential. The Food and Drug Administration approved Lecanemab and aducanumab as the first therapy with disease-modifying effects in 2023. Lecanemab has shown efficacy in several trials, with the potential to improve cognition in AD patients. In this article, we will discuss the treatment options for AD, emphasizing the newly approved monoclonal antibodies and their prospects.

## Editorial

Alzheimer’s disease (AD) is the most prevalent neurodegenerative disease in the world, with approximately 55 million patients worldwide and around 6.7 million, aged 65 or above, living in the USA in 2023. It is expected that by 2050, the number of Americans, 65 years or older affected by Alzheimer’s may increase to about 12.7 million. AD has a huge financial toll on the US healthcare system; AD will cost the nation around $345 billion in 2023, while the lifetime care of patients with AD is estimated at $392,874 [1]. AD dementia leads to progressive cognitive and functional decline, affecting the performance of daily life activities. The pathophysiological features involve the deposition of soluble and insoluble extracellular amyloid plaques and hyperphosphorylated tau proteins, leading to the deposition of intracellular neurofibrillary tangles, resulting in loss of neurons and their connections in the brain, resulting in brain atrophy [2].

Before 2023, few traditional pharmacotherapies were approved by the Food and Drug Administration for only symptomatic AD treatment. These include the cholinesterase inhibitors (ChEIs), which include galantamine, rivastigmine, and donepezil, and the non-competitive NMDA receptor antagonists (NMDARAs), memantine. These drugs collectively are used for symptomatic improvement in cognition and behavioral symptoms. The ChEIs act by inhibiting the breakdown of acetylcholine, thus increasing its availability and duration of action. While NMDARAs act by blocking the glutamate receptors, thus preventing synaptic dysregulation and neuron death. These drugs are also used together for enhanced beneficial effects [2]. Recently, the FDA approved the first disease-modifying monoclonal antibodies, lecanemab and aducanumab. These are disease-modifying treatments for AD that can decrease the progression of the disease. These monoclonal antibodies are directed against insoluble amyloid beta proteins and aggregated protofibrils [3]. They play a significant role in removing the amyloid plaques, decreasing amyloid PET detection [4].

Lecanemab was approved by the FDA in 2023. The researchers evaluated lecanemab efficacy by comparing it to the ChEIs and NMDARAs in different trials. A double-blind, placebo-controlled trial of 856 patients (Lecanemab:609-Placebo:245) with mild dementia, cognitive impairment, and amyloid plaques revealed significant results. Patients received 10 mg/kg lecanemab every two weeks. Analysis at 18 months showed that the lecanemab was associated with less clinical decline as compared to the placebo, along with clearance of amyloid plaques with lecanemab. The clinical decline measured by the ADCOMS scale was only 27% in the lecanemab group compared to 30% in the placebo group. Moreover, the ADAS-Cog14 scale showed 56% less decline in the lecanemab group than 47% in the placebo group [3]. Another phase 3 multicenter clinical trial was conducted with 1,795 mild AD patients (Lecanemab:898- Placebo-897). Results over an 18-month period revealed that the progression of the illness was slowed by 27% in the lecanemab group compared to the placebo group. The mean Clinical Dementia rating- the sum of boxes (CDR-SB) at baseline was approximately 3.2 in both groups and after treatment, change was 1.21 with lecanemab and 1.66 with placebo at 18 months. At the start of the study, the mean amyloid level was 77.92 and 75.03 centiloids in the lecanemab and the placebo group, respectively. The results of the 18-month analysis showed a significant difference in the mean change with −55.48 centiloids in the lecanemab group compared to 3.64 centiloids in the placebo group. Similarly, the mean change in the ADAS-cog14 score, at 18 months, was 4.14 in the lecanemab group compared to 5.58 in the placebo group [5].

The studies have shown a few side effects of lecanemab. Some side effects were minor such as headache, infusion-related reactions, and a rare incidence of falls. Some patients developed a serious side effect which is amyloid-related imaging abnormalities (ARIA), including brain edema (ARIA-E) or intracerebral hemorrhage (ARIA-H). The incidence of ARIA-H was 17.3% with lecanemab and 9.0% with placebo. A total of 12.6% of patients in the lecanemab group had ARIA-E as compared to 1.7% in the placebo group. Out of these 12.6%, only 2.8% had symptomatic ARIA-E. ARIA-E presented as temporary swelling, confusion, dizziness, and vision changes. In some patients, ARIA-E progressed to brain edema along with seizures and neurological deficits. Studies have recommended that there should be careful monitoring for ARIA especially during the first 14 weeks of treatment with lecanemab [5]. Moreover, patients with homozygous ApoE ε4 allele had more incidences of ARIA; therefore, it is always indicated for genotyping of ApoE ε4 for anyone considering taking lecanemab. Patients taking anticoagulation should take lecanemab with caution because of the increased risk of intracerebral hemorrhage [5].

The other monoclonal antibody, aducanumab, was approved by the FDA in 2021 by accelerated pathway for cognitive improvement of AD. Aducanumab is an IgG1 monoclonal antibody that targets Aβ amyloid proteins in the brain. Two studies, ENGAGE and IMERGE enrolled 3285 patients between 50 and 85 years of age with mild dementia. They were stratified on ApoE ɛ4 carrier status and randomized on the basis of low-dose Aducanumab, high-dose Aducanumab, and placebo. The results were calculated at 78 weeks. The baseline CDR-SB had a score of 0.5. The CDR-SB scores in the low-dose group and the high-dose group went down to 0.18 and 0.03 compared to placebo in the ENGAGE trial, respectively. In the EMERGE trial, the treatment group showed -0.26 and -0.39 CDR-SB scores in low-dose and high-dose sub-groups, respectively. The treatment group responded with a 22% relative reduction in the high dose aducanumab group compared to the placebo in the IMERGE trial. The EMERGE revealed a statistically significant change in all primary or secondary clinical endpoints, while the ENGAGE could not meet its endpoints [4,6]. A few side effects of aducanumab included headache, diarrhea, falls, ARIA-E and ARIA-H. The risk of ARIA was greater in patients homozygous for ApoE ɛ4 therefore their genotyping is important before the start of treatment. The risk of ARIA-E was at 35.2% in the high-dose treatment group compared to 2.7% in the placebo group (Table 1) [4].

**Table 1:** Treatment options for Alzheimer's disease [2-4]

Drug	Type	Mechanism of Action	Efficacy	Side Effects
Cholinesterase inhibitor (galantamine, rivastigmine, donepezil)	Enzyme inhibitor.	Decrease the breakdown of acetylcholine, leading toincreased availability.	Temporary symptomatic improvement.	Headaches, dizziness, vomiting, loss of appetite, diarrhea, muscle cramps, and asthenia.
Glutamate NMDA receptor antagonist (memantine)	Receptor blocker.	Blockage of glutamate NMDA receptors leading to decreased action of glutamate.	Neuroprotective, used in combination with ChEIs for symptomatic relief.	Dizziness, Headaches, constipation, confusion, and shortness of breath.
Lecanemab	Monoclonal antibody.	Removal of amyloid plaques in the brain and amyloid protofibrils.	Modification of disease pathology leading to symptomatic improvement.	Headaches, ARIA (cerebral edema and intracerebral hemorrhage), and Infusion-related reactions.
Aducanumab	Monoclonal antibody.	Removal of amyloid plaques in the brain and amyloid protofibrils.	Modification of disease pathology leading to symptomatic improvement.	Headache, dirrahea, falls, ARIA-E and ARIA-H.

AD impairs the daily life functioning of patients. With the already available treatment options, AD patients used to experience symptomatic improvements. However, with the approval of the disease-modifying treatments lecanemab and aducanumab, AD patients have developed hopes to finally treat their disease on a pathological level, along with significant improvements in cognition and daily life functions. Lecanemab and aducanumab have shown promising results and have been proven beneficial when initiated in mild dementia and cognitive impairment with the confirmatory presence of amyloid beta plaques. These monoclonal antibodies are redefining AD care because of their disease-modifying mechanism. They can potentially improve AD patients' long-term quality of life so they can function independently.
